# Investigating the interrelationships among food habits, sports nutrition knowledge, and perceived barriers to healthy eating: a study of adolescent swimmers

**DOI:** 10.3389/fnut.2024.1381801

**Published:** 2024-05-14

**Authors:** Walaa AlKasasbeh, Sofia Akroush

**Affiliations:** Department of Physical and Health Education, Faculty of Education Sciences, Al-Ahliyya Amman University, Al-Salt, Jordan

**Keywords:** food habits, sports nutrition knowledge, perceived barriers, healthy eating, adolescents swimmers

## Abstract

This cross-sectional study aims to explore the relationships between food habits, perceived barriers to healthy eating, and sports nutrition knowledge among adolescent swimmers. The study focuses on understanding how these factors interact and influence dietary choices in young athletes. A cohort of 52 adolescent swimmers aged 12–18 from Al Hussein Youth Club in Al-Hussein Sport City participated in the study. Data were collected through surveys assessing food habits, perceived barriers to healthy eating, and sports nutrition knowledge. Pearson Correlation analysis was employed to examine associations between variables, and stepwise regression analysis was used to identify predictors of food habits. The analysis revealed a significant positive association between food habits and sports nutrition knowledge (*r* = 0.393, *p* = 0.004). Knowledge emerged as a significant positive predictor of food habits (*β* = 0.393, *p* = 0.004), highlighting the influential role of sports nutrition knowledge in shaping the dietary choices of adolescent swimmers. However, the introduction of Barriers lacked significance, and individual predictors did not reach statistical significance. These findings underscore the importance of targeted interventions aimed at enhancing nutritional education among adolescent swimmers. Understanding the interplay between knowledge, barriers, and food habits provides valuable insights into the complex dynamics that influence the dietary choices of young athletes. Addressing these factors through tailored educational programs can promote healthier eating habits and optimize performance among adolescent swimmers. This study highlights the critical role of sports nutrition knowledge in shaping the dietary behaviors of adolescent swimmers. By addressing knowledge gaps and overcoming perceived barriers, targeted interventions can help improve food habits and enhance the overall health and performance of young athletes.

## Introduction

Insufficient understanding of nutrition among athletes has the potential to impact their performance (1, 2). It is crucial for adolescent athletes to embrace a healthy eating regimen to adequately fulfill their nutritional requirements, foster well-being, and optimize their athletic performance (3, 4). While sports organizations worldwide emphasize the significance of continuous nutritional education for young athletes, coaches, and parents (5, 6), the extent of nutrition education among adolescent athletes, particularly swimmers, remains inadequately explored.

Nonetheless, the necessity for athlete education is apparent, as evidenced by a study involving elite athletes, a substantial proportion of them were aged under 18 years. These athletes exhibited lower scores in the section about ‘nutrient sources’, as compared to individuals who are not athletes (7). Additionally, a comprehensive analysis of nutrition knowledge (NK) among competitive, recreational, or elite athletes aged 13 and above revealed that while athletes’ understanding of nutrition was on par with or superior to that of non-athletes, it lagged behind when compared to non-athlete comparison groups (8). Several research studies have demonstrated inadequate compliance of athletes with dietary guidelines, coupled with insufficient consumption of essential micronutrients (9, 10). Enhancing the Food habits (FH) of adolescent swimmers (ASs) could potentially involve bolstering their understanding of nutrition.

The Consensus Statement on ‘Nutrition for Swimming’ from The International Journal of Sport Nutrition and Exercise Performance proposes achieving this objective by implementing a carefully designed nutrition education program. This initiative is intended to systematically enhance participants’ understanding of nutrition principles (11). It is widely acknowledged that achieving peak athletic performance can be significantly influenced by optimal nutrition (12). Nonetheless, several obstacles have the potential to impede athletes from attaining the best possible dietary practices. These challenges encompass constraints such as inadequate time and kitchen facilities to cook meals, limited financial means, deficient abilities in meal planning and preparation, and demanding travel schedules (13).

Studies on nutrition education have echoed comparable outcomes, showcasing its efficacy in enhancing individuals’ understanding of food, their dietary behaviors, and their mindfulness about nutrition. These results underscore the necessity and effectiveness of nutrition education in augmenting knowledge about nutrition, self-confidence in making dietary choices, and fostering favorable shifts in perceptions (14).

Studies involving children have indicated that interventions encompassing multiple components and involving parental participation have demonstrated the greatest success in enhancing FH (15). However, nutrition education programs of this nature are relatively underexplored in the context of adolescent athletes. Only a few isolated cases have yielded mixed outcomes in this regard (16, 17). Despite research indicating that numerous athletes tend to underestimate their nutrient requirements (18) and frequently fall short of meeting the nutrition guidelines essential for optimal performance (19–21), limited attention has been given to exploring the potential barriers to healthy eating among ASs.

During the adolescent phase, the nutritional demands for sports involvement become more complex due to the simultaneous requirements of growth, physical development, and psychological maturation. Inadequacies in nutrition during this period can lead to enduring repercussions extending into adulthood (22). Adolescence serves as a crucial juncture for establishing dietary patterns (22). Consequently, special attention is needed for the FH and NK of teenage athletes (23).

The dietary routines of young swimmers can be influenced by their substantial time commitment to clubs and training centers, necessitating meals outside their homes. Frequently, they are accountable for their food choices, which can pose challenges in maintaining a nutritious and balanced diet to counterbalance the energy expended in their athletic pursuits. Considering the level of autonomy adolescent athletes possess over their diet and physical activities, having a grasp of nutrition can play a pivotal role in cultivating appropriate eating behaviors in this group. Nevertheless, research in diverse cultural settings has exposed gaps in athletes’ nutritional understanding, encompassing both general nutritional aspects (24) and comprehension of their sport-specific requirements (23, 25). These knowledge deficiencies can be amplified by misconceptions about dietary practices (23) that may stem from superstitions or unreliable advice.

In order to foster more healthful dietary practices among adolescent athletes, it is crucial to confront these hurdles and equip them with precise nutritional instruction. This instruction should not solely concentrate on fundamental nutritional principles but also extend to the specific dietary prerequisites linked to their sport. This approach empowers athletes to make knowledgeable decisions that bolster their performance and overall well-being. Given the demanding schedules of ASs, which involve training and school responsibilities, it becomes crucial to comprehend the barriers they face when making nutritional choices.

The aim of this study was to explore the correlations between FH, perceived obstacles to maintaining a healthy diet, and the level of SNK among ASs. The study examined potential links between the FH of ASs and their PBHE to adopting a healthy diet.

The first hypothesis proposes that there is a positive association between the quality of FH and the level of SNK among ASs, resulting in a lower PBHE. The second hypothesis suggests that various demographic factors, including swimmer category, gender, training experience, and BMI, may act as moderators in the relationship between FH, PBHE, and SNK among ASs. It is postulated that these demographic variables influence how the aforementioned relationships manifest.

## Materials and methods

### Participants

In the conducted study, a cross-sectional design was implemented to explore the interplay between dietary habits, PBHE, and SNK among ASs associated with Al Hussein Youth Club in Al-Hussein Sport City. The participant recruitment process commenced in August 2023, coinciding with researchers’ visits to the Olympic swimming pool in Al-Hussein Sport City. During these visits, 71 swimmers aged between 12 and 18 years, classified into the second, third, fourth, and fifth categories of both genders according to the International Swimming Federation (FINA), were informed about the research’s purpose and invited to volunteer. A total of 52 swimmers (73.2%) willingly participated, with assurances of confidentiality and no incentives provided. Informed consent from participants and parent/guardian assent was obtained prior to their involvement in the study.

The data collection phase occurred concurrently with the Jordanian Clubs Swimming Championship at the Olympic Pool in Al-Hussein Sport City, spanning from August 23, 2023, to August 27, 2023. While coaches did not partake in the study, they, along with researchers, were available to assist participants in clarifying questionnaire-related queries. The completion of surveys took an average of 45 min. The study protocol received approval from NAMA Strategic Intelligence Solutions (No. M-SH/1/523).

The criteria for inclusion and exclusion from the group in the conducted study were defined to ensure the relevance and reliability of the research findings. Inclusion criteria encompassed participants aged between 12 and 18 years who were affiliated with Al Hussein Youth Club in Al-Hussein Sport City and fell into the second, third, fourth, or fifth categories according to the International Swimming Federation (FINA). Additionally, voluntary participation and informed consent were essential prerequisites for inclusion. Conversely, exclusion criteria encompassed individuals outside the specified age range, those not associated with the mentioned club, and those not classified within the designated swimmer categories by FINA. These criteria were established to maintain the study’s focus on a specific population group namely, ASs from Al Hussein Youth Club and to mitigate potential confounding variables while adhering to ethical guidelines regarding informed consent and participant confidentiality.

This diverse study population, as evidenced by the distribution of participants across various demographic variables, contributes to a comprehensive understanding of the research objectives. The majority of participants fell into the third category, representing 40.4%, followed by the second category at 13.5%. Gender distribution revealed a slight majority of male participants (53.8%), while 46.2% identified as female. Participants’ Body Mass Index (BMI) varied, with the largest proportion falling within the 25^th^ Percentile range (44.2%). Experience levels were also diverse, with 5–7 years being the most common bracket at 38.5%.

### Procedures

The procedures employed for data collection in this study involved the use of a self-administered printed questionnaire to gather information on participants’ demographic details, PBHE, SNK, and FH. Specifically, for ASs, the research team conducted direct measurements of height and weight using the Health-o-Meter portable digital floor scale. To standardize body weight data collection, swimmers were instructed to wear a swimsuit.

Participants’ height and weight data were utilized to calculate body mass index (BMI) in kilograms per square meter (kg/m^2^). Studies have shown a strong correlation between self-reported and measured BMIs (26). Subsequently, the height and weight data underwent comparison with the Centers for Disease Control (CDC) growth charts to identify any instances of self-reported values that deviated excessively from the norm (27).

### Instruments

#### Perceived barriers to healthy eating (PBHE)

A set of questions addressing barriers to maintaining a healthy diet was adapted from a prior study focusing on barriers to weight management (28). Participants were presented with five response options: ‘strongly disagree,’ ‘disagree,’ ‘neither agree nor disagree,’ ‘agree,’ and ‘strongly agree’. Each option was assigned a numerical value ranging from 1 to 5, indicating higher barriers with increasing values. However, in the section covering social and environmental barriers, a modification was made to the original paragraph. Instead of ‘Not having time to prepare or eat healthy foods due to ‘job,” the revised statement reads, ‘Not having time to prepare or eat healthy foods due to school.’ This adjustment is more applicable to adolescents who are primarily engaged in education rather than work. Similarly, the phrase ‘Not having time to prepare or eat healthy foods because of my family commitment’ was revised to ‘Not having time to prepare or eat healthy foods because of my commitment to training’. This modification is made to clarify that this category pertains to other obligations, such as training, whereas family commitments are more relevant to adults. The internal consistency of measurement scale was assessed based on a survey sample of 25 participants. The PBHE Scale, comprising 11 items, demonstrated a moderate level of reliability with a Cronbach’s Alpha of 0.724. These reliability statistics suggest that the items within scale consistently measure the intended constructs, providing a reliable foundation for further analyses in exploring PBHE within the surveyed sample (see Table 1).

**Table 1:** Internal consistency assessment of scales.

Scale	Cronbach’s Alpha	Number of items
Perceived barriers to healthy eating (PBHE)	0.724	11
Food habits (FH)	0.724	13
Sports nutrition knowledge (SNK)	0.731	59

#### Sports nutrition knowledge (SNK)

The survey utilized to assess the nutritional knowledge (NK) of athletes was the NK for Athletes survey, chosen for its validated suitability for this specific purpose. This survey, having undergone rigorous validation procedures, offers attributes such as minimal time burden, updated content, and user-friendliness, particularly suitable for early adolescents. The survey comprises 59 items that cover various aspects of sports nutrition, including macronutrients, micronutrients, hydration, and the frequency of food intake (29). Participants were asked to provide responses to these items, and each correct answer was awarded +1 point, incorrect answers were given a score of −1 point, and unanswered items received 0 points. Higher scores on the survey indicated a higher level of nutrition knowledge, with the maximum achievable score being 59 points. The SNK Scale, a more extensive measure with 59 items, maintained a moderate level of internal consistency, reflected by a Cronbach’s Alpha of 0.731. These reliability statistics suggest that the items within scale consistently measure the intended constructs, providing a reliable foundation for further analyses in exploring SNK within the surveyed sample (see Table 1).

#### Food habits (FH)

Food habits: encompassing a set of 14 inquiries. One question has been excised: Is wine or beer commonly consumed during meals? This omission stems from the study’s focus on adolescents aged 12–18, making the question incongruous for this demographic. Consequently, the total number of questions stands at 13.

The aim of this segment was to examine the FH of adolescents, with a particular focus on aspects like the composition of breakfast, frequency of daily meals, regular intake of fruits and vegetables, and consumption patterns of soft beverages.

Seven of the questions had the following response categories: “always,” “often,” “sometimes,” and “never.” Conversely, the remaining six questions employed distinct structures featuring four response categories. Each response was allocated a score between 0 and 3, where the highest score indicated the healthiest choice and the lowest denoted the least healthy. The cumulative score attainable for this section amounted to 39. The FH Scale, consisting of 13 items, exhibited comparable reliability with a Cronbach’s Alpha of 0.724. These reliability statistics suggest that the items within scale consistently measure the intended constructs, providing a reliable foundation for further analyses in exploring FH within the surveyed sample (see Table 1).

### Statistical analyses

The study employed a variety of statistical analyses to thoroughly examine different aspects of the collected data. Initially, an internal consistency assessment was conducted using Cronbach’s Alpha, a widely accepted measure for evaluating scale reliability. The study employed descriptive statistics and categorical analysis, presenting counts and row percentages for various categorical variables related to the scales. Furthermore, correlation analysis was applied with Pearson Correlation coefficients calculated to explore relationships between different scales. Finally, a stepwise regression analysis was performed to identify predictors influencing food habits. Together, these analyses provide a comprehensive and insightful examination of scale reliability, inter relationships between variables, and factors influencing FH within the surveyed sample.

## Results

Table 2 presents a breakdown of categorical variables related to different scales, each classified into low, moderate, and high levels. The categories include food habits.cat, personal Barriers.cat, social Barriers.cat, environmental Barriers.cat, barriers.cat, and Sports Nutrition Knowledge.cat. The counts and row percentages for each level are provided, offering insights into the distribution of participants across the specified categorical variables.

**Table 2:** Categorical variables describing scales.

	Low		Moderate		High	
	Count	Row *N* %	Count	Row *N* %	Count	Row *N* %
Food habits.cat	14	26.9%	26	50.0%	12	23.1%
Personal barriers.cat	18	34.6%	21	40.4%	13	25.0%
Social barriers.cat	16	30.8%	27	51.9%	9	17.3%
Environmental barriers.cat	15	28.8%	27	51.9%	10	19.2%
Perceived barriers.cat	15	28.8%	27	51.9%	10	19.2%
Sports nutrition knowledge.cat	13	25.0%	28	53.8%	11	21.2%

Table 3 explores the interconnections among various scales, specifically examining the relationships between food habits, personal barriers, social barriers, environmental barriers, overall barriers, and knowledge. The table presents Pearson Correlation coefficients along with corresponding two-tailed significance levels (Sig.). Additionally, the sample size (*N*) for each correlation is provided, enhancing the understanding of statistical relationships based on data collected from 52 participants. Notably, a significant positive correlation is evident between the ‘Food Habits’ and ‘Knowledge’ scales, indicated by a Pearson Correlation coefficient of 0.393 and a noteworthy two-tailed *p*-value of 0.004.

**Table 3:** Correlations between scales-investigating Pearson correlations and significance levels.

		Food. Habits
Personal barriers	Pearson Correlation	−0.097
	Sig. (2-tailed)	0.492
	*N*	52
Social barriers	Pearson Correlation	−0.209
	Sig. (2-tailed)	0.136
	*N*	52
Environmental barriers	Pearson Correlation	−0.097
	Sig. (2-tailed)	0.492
	N	52
Perceived barriers	Pearson Correlation	−0.183
	Sig. (2-tailed)	0.193
	*N*	52
Sports nutrition knowledge(SNK)	Pearson Correlation	0.393^**^
	Sig. (2-tailed)	0.004
	*N*	52

**Statistical significance *p* < 0.05.

Table 4 presents a comprehensive overview of a stepwise regression analysis aimed at identifying predictors influencing food habits. In the initial step, SNK emerges as a statistically significant positive predictor (*β* = 0.393, *p* = 0.004), indicating a positive association with FH. However, introducing PBs in Step 2 results in a statistically significant positive relationship (*β* = −0.173, *p* = 0.187). In the absence of a statistical indicator of barriers to eating healthy food, the positive association between SNK and FH seems to lead to a reduction in barriers to eating healthy food.

**Table 4:** Stepwise regression analysis of predictors influencing FH.

Variables	Step 1			Step 2			Step 3		
	*B*	*β*	*p*	*B*	*β*	*p*	*B*	*Β*	*p*
Sports nutrition knowledge(SNK)	0.136	0.393	0.004	0.134	0.389	0.004	0.124	0.360	0.010
Perceived barriers(PB)	–	–	–	−0.095	−0.173	0.187	−0.104	−0.188	0.163
Category	–	–		–	–	–	0.273	0.103	0.481
Gender	–	–	–	–		–	0.284	0.041	0.785
BMI	–	–	–	–	–	–	−0.665	−0.164	0.223
Experience	–	–	–	–	–	–	0.040	0.010	0.949
R2	0.155			0.184			0.250		
Model fit	*F* = 9.153 *p* = 0.004			*F* = 5.543 *p* = 0.007			*F* = 2.173 *p* = 0.063		
R2 change				0.029			0.066		

Statistical significance *p* < 0.05.

Moving to Step 3, additional predictors, including Category, Gender, BMI, and Experience, are introduced. However, none of these individual predictors demonstrates a statistically significant relationship with FH at this step Category (*β* = 0.103, *p* = 0.481), Gender (*β* = 0.041, *p* = 0.785), BMI (*β* = −0.164, *p* = 0.223), and Experience (*β* = 0.010, *p* = 0.949).

Despite the lack of individual predictor significance, the overall model remains statistically significant across all steps (Step 1: *F* = 9.153, *p* = 0.004; Step 2: *F* = 5.543, *p* = 0.007; Step 3: *F* = 2.173, *p* = 0.063). The R2 values indicate the proportion of variance explained, progressively increasing from 0.155 in Step 1 to 0.250 in Step 3. The incremental change in R2 is 0.029 in Step 2 and 0.066 in Step 3, suggesting notable enhancements in the model’s explanatory power upon the inclusion of predictors.

## Discussion

This study investigated the correlations between FH, PBHE, and the SNK among ASs, while also considering the moderating role of demographic factors in connecting FH, PB, and SNK. Two hypotheses are proposed: the first suggests a positive association between FH and SNK, leading to a lower perception of barriers; the second proposes that demographic factors may moderate the relationships between FH, PBHE, and SNK among ASs.

Nutrition understanding significantly impacts athletes’ performance, emphasizing the importance of a healthy eating regimen to meet nutritional requirements and optimize athletic performance. Despite global recognition of the importance of continuous nutritional education for young athletes (5, 6), the precise exploration of fundamental dietary patterns among ASs remains insufficient within the existing body of literature. This study aims to fill this gap by examining SNK, FH, and PBHE among ASs, particularly in Jordanian athletes.

The study revealed a significant positive correlation between FH and SNK, indicating that an increase in SNK among ASs correlates with an improvement in the quality of their FH.

This aligned with previous research showing a positive association between nutrition knowledge and practices among adolescent athletes (30–32), the positive correlation between dietary intake and SNK among Adolescent Soccer Players supports this finding (33). Notably, our research outcomes are contrasts with a study demonstrating that possessing knowledge does not always result in the adoption of good dietary practices (34). The positive association between SNK and FH emphasizes the pivotal role of education in shaping athletes’ dietary choices (14, 35). Educational interventions targeting SNK enhancement could lead to positive changes in the dietary practices of young athletes (36). For example, Foo et al. (37) conducted a nutrition education intervention among highly trained ASs, showing a significant improvement in SNK scores, indicating the positive influence of targeted education on athletes’ knowledge.

The results offer a nuanced perspective on the factors influencing food habits, shedding light on the relationships between SNK, PBs, and various demographic factors. Initially, a statistically significant positive association between SNK and FH (*β* = 0.393, *p* = 0.004) aligns with existing literature emphasizing the positive impact of nutrition knowledge on dietary choices (31, 38, 39). This underscores the role of education in fostering healthier eating habits among participants.

The introduction of PBs in Step 2 of the analysis reveals a noteworthy finding. The statistically significant positive relationship between PBs and FH suggests that as SNK increases, there is a concurrent reduction in personal barriers to healthy eating among ASs. This unexpected relationship can be seen as a positive outcome, indicating that higher knowledge levels empower individuals to overcome perceived obstacles, fostering the adoption of healthier dietary habits. Further exploration into the specific nature of these PBs and how increased knowledge contributes to their mitigation is warranted like targeted education initiatives that focus on increasing nutritional knowledge and practical skills have the potential to effectively address barriers to healthy eating. By empowering individuals to make informed choices, develop essential cooking skills, and cultivate a positive mindset toward nutrition, these initiatives can facilitate long-term behavior change and promote healthier lifestyles among athletes. The observed reduction in PBs with enhanced SNK underscores the potential effectiveness of targeted education initiatives. Studies by Brauman et al. (30) have identified significant barriers to healthy eating, such as lack of time, easy access to unhealthy foods, cost, lack of knowledge, and cooking skills. Additional research has shown that adolescents identify a lack of willpower and a hectic lifestyle as primary obstacles to embracing a healthy diet (33). These findings highlight the positive impact of targeted education initiatives in addressing barriers to healthy eating among athletes (40). By providing athletes with the knowledge and skills to navigate challenges such as time constraints, limited access to healthy foods, and financial considerations, educational interventions contribute to creating an environment conducive to healthier dietary choices (41).

In Step 3 of the analysis, additional predictors including category, gender, BMI, and experience are introduced. Despite their inclusion, none of these individual predictors demonstrates a statistically significant relationship with FH in this step. This lack of significance suggests that, in the context of this study, these demographic factors may not independently influence participants’ dietary behaviors. However, it is crucial to acknowledge the potential interplay and cumulative effect of these factors, which might collectively contribute to shaping FH.

The overall model’s persistence in statistical significance across all steps highlights the collective explanatory power of the included predictors. The increasing R-squared (R^2^) values signify the progressively improved ability of the model to explain the variance in food habits. The incremental change in R2 between steps indicates notable enhancements in the model’s explanatory power upon the inclusion of predictors, reinforcing the notion that a combination of SNK, PB, and demographic factors collectively contributes to the understanding of FH among participants. In accordance with our findings, another study also supported the results presented in this paper by detecting no differences based on sex or category (42). Similarly, a study found no significant correlations between sex, BMI, SNK, and FH (43). Additionally, another study showed no differences between SNK and gender (18). Conversely, a study validated that knowledge was notably linked to the age, gender, and duration of sports training among participants. Meanwhile, only age and BMI exhibited significant associations with FH (44).

The study’s identification of a significant positive relationship between PBHE and FH, coupled with the unexpected reduction in personal barriers as SNK increases, underscores the potential effectiveness of targeted educational initiatives. The results suggest that enhancing knowledge empowers individuals to overcome obstacles, fostering the adoption of healthier dietary habits. Further exploration into the specific nature of these barriers and how increased knowledge contributes to their mitigation could provide valuable insights for future interventions. The introduction of demographic factors, including category, gender, BMI, and experience, did not show individual significance in influencing food habits. However, the study acknowledges the potential cumulative effect and interplay of these factors, emphasizing the collective explanatory power of SNK, PB, and demographics in understanding participants’ FH. In light of these findings. The head coach, along with assistant coaches, plays a crucial role in reinforcing proper dietary practices among swimmers. This comprehensive approach extends beyond just working with the athletes; it involves engaging with both the swimmers and their parents. This strategy emphasizes that the SNK acquired is not just theoretical but is actively implemented in the athletes’ daily lives. Numerous studies underscore the importance of involving parents in the nutritional education process. This inclusion recognizes the influential role parents play in shaping their child’s dietary habits, ensuring a holistic approach to nutrition education and the major parental role in strengthening NK among adolescent competitive swimmers (45). This inclusion recognizes the influential role parents play in shaping their child’s dietary habits, ensuring a holistic approach to nutrition education and the major parental role in strengthening nutrition knowledge among adolescent competitive swimmers (46). Additionally, coaches act as mentors and facilitators in the athletes’ journey toward optimal nutrition, providing guidance and oversight. This involvement serves not only to educate but also to reinforce positive nutritional behaviors. By actively participating in tracking food habits, coaches contribute to the overall well-being of the athletes under their guidance, fostering a culture of health and performance. However, it’s crucial to acknowledge that athlete perceptions of coaches helping them track food intake can be highly controversial. Some athletes may feel subverted or like they are being too closely monitored by coaches, which can lead to negative outcomes such as developing a strained relationship with food and body image issues. This integrated approach, encompassing both parental involvement and coach-led monitoring, aligns with the broader sports community’s understanding of the interconnected factors influencing athletes’ nutritional choices and performance outcomes.

It is evident that targeted education initiatives play a crucial role in addressing barriers and fostering healthier dietary choices among young athletes. Crafting suitable nutrition education strategies using online resources and mobile applications can effectively bolster both nutritional knowledge and practices among athletes (47). Research indicates that mobile applications are especially proficient in augmenting nutrition knowledge (48). By integrating mobile applications, there exists substantial potential to elevate nutritional understanding (49) consequently fostering healthier dietary habits and advancing nutritional knowledge even further (50). Our study calls for continued efforts in providing athletes with the knowledge and skills to navigate challenges, such as time constraints, limited access to healthy foods, and financial considerations. Moreover, our study emphasizes the need for tailored educational programs to address the specific context of adolescent swimmers, contributing to the optimization of nutritional practices and, consequently, athletic performance.

It’s imperative to acknowledge several potential limitations associated with the dataset utilized in our study. Firstly, the sample size of the dataset may be limited, which could hinder the generalizability of our findings to a broader population of athletes. Secondly, our study may have employed a cross-sectional design, gathering data at a singular time point. This approach limits our ability to establish causality or track changes in dietary behaviors over time. Lastly, the conclusions drawn from our study may be specific to the context of adolescent swimmers. Consequently, the generalizability of our results to other athlete populations or different settings may be constrained. Recognizing these potential limitations is crucial for interpreting the implications of our study accurately and for guiding future research endeavors aimed at addressing the complexities of nutrition education among athletes.

## Conclusion

In conclusion, this study sheds light on the relationships among FH, PBHE, and SNK within a cohort of 52 ASs aged 12–18 from Al Hussein Youth Club in Al-Hussein Sport City. The significant positive association between FH and SNK underscores the importance of SNK in shaping the dietary choices of young athletes. However, the lack of significance in introducing PB and individual predictors suggests that the influence of barriers and demographic factors may not be as straightforward in determining FH in this specific population. While individual predictors did not achieve statistical significance, the overall model remained significant, emphasizing the multifaceted nature of factors influencing FH among ASs. These findings contribute valuable insights into the nuanced dynamics of nutritional behaviors in ASs, highlighting the need for targeted interventions to enhance nutritional education. Some targeted interventions that could be considered include, Firstly, Implementing nutrition education workshops specifically tailored to the needs and challenges of adolescent swimmers, addressing topics such as meal planning, nutrient timing, and hydration strategies. Secondly, providing access to online resources and mobile applications designed to enhance nutrition knowledge and promote healthy eating habits among adolescent swimmers. Additionally, collaborating with nutritionists or dietitians to offer personalized dietary counseling and guidance to athletes, considering their individual nutritional needs and goals. Lastly, Incorporating nutrition education into the overall training program for adolescent swimmers, emphasizing the importance of proper nutrition for performance, recovery, and overall health. The study advocates for the development of tailored educational programs that address the specific nutritional needs and challenges faced by ASs. By enhancing SNK, interventions can empower these athletes to make informed dietary choices, ultimately optimizing their performance and overall well-being. Future research endeavors should delve deeper into the diverse factors contributing to FH in adolescent athletes, considering contextual and individual variables that may influence nutritional behaviors. Such insights will aid in the formulation of more precise and effective interventions, fostering a culture of healthy FH among ASs. However, it’s important to note that with a small sample size, the findings of the study may not be representative of the broader population of ASs. The demographics, experiences, and health behaviors of the participants might not be reflective of other groups, limiting the generalizability of the results.

## Data availability statement

The raw data supporting the conclusions of this article will be made available by the authors, without undue reservation.

## Ethics statement

Written informed consent was obtained from the minor(s)' legal guardian/next of kin for the publication of any potentially identifiable images or data included in this article.

## Author contributions

WA: Conceptualization, Formal analysis, Investigation, Methodology, Supervision, Validation, Writing – original draft, Writing – review & editing. SA: Data curation, Writing – original draft.

## References

[1] Amawi AT Moualla DS Alshuwaier GO Al-Nuaim AA Bursais AK Aljaloud KS. Knowledge and attitude of dietary supplements among Arab Olympic athletes and coaches in preparation program for Tokyo 2020 Olympic games. Int J Hum Mov Sports Sci. (2023) 11:368–77. doi: 10.13189/saj.2023.110214

[2] Amawi A Alshuwaier G Alaqil A Alkasasbeh WJ Bursais A Al-Nuaim A. Exploring the impact of dietary factors on body composition in elite Saudi soccer players: a focus on added sugars, salt, and oil consumption. Int J Hum Mov Sports Sci. (2023) 11:1305–12. doi: 10.13189/saj.2023.110614

[3] Desbrow B McCormack J Burke LM Cox GR Fallon K Hislop M. Sports dietitians Australia position statement: sports nutrition for the adolescent athlete. Int J Sport Nutr Exerc Metab. (2014) 24:570–84. doi: 10.1123/ijsnem.2014-0031, PMID: 24668620

[4] Rodriguez NR DiMarco NM Langley S. Dietitians of Canada, and the American College of Sports Medicine: nutrition and athletic performance. J Am Diet Assoc. (2009) 109:509–27. doi: 10.1016/j.jada.2009.01.005, PMID: 19278045

[5] Mountjoy M Armstrong N Bizzini L Blimkie C Evans J Gerrard D. IOC consensus statement: “training the elite child athlete”. Br J Sports Med. (2008) 42:163–4. doi: 10.1136/bjsm.2007.044016 18048429

[6] Meyer F O’Connor H Shirreffs SM. Nutrition for the young athlete. J Sports Sci. (2007) 25:S73–82. doi: 10.1080/02640410701607338 18049985

[7] Spendlove JK Heaney SE Gifford JA Prvan T Denyer GS O’Connor HT. Evaluation of general nutrition knowledge in elite Australian athletes. Br J Nutr. (2012) 107:1871–80. doi: 10.1017/S0007114511005125, PMID: 22018024

[8] Heaney S O’Connor H Michael S Gifford J Naughton G. Nutrition knowledge in athletes: a systematic review. Int J Sport Nutr Exerc Metab. (2011) 21:248–61. doi: 10.1123/ijsnem.21.3.248 21719906

[9] Martínez S Pasquarelli BN Romaguera D Arasa C Tauler P Aguiló A. Anthropometric characteristics and nutritional profile of young amateur swimmers. J Strength Cond Res. (2011) 25:1126–33. doi: 10.1519/JSC.0b013e3181d4d3df, PMID: 20838252

[10] de Sousa EF Da Costa THM Nogueira JAD Vivaldi LJ. Assessment of nutrient and water intake among adolescents from sports federations in the Federal District. Br J Nutr. (2008) 99:1275–83. doi: 10.1017/S0007114507864841, PMID: 18053313

[11] Burke L. Nutrition for swimming. Int J Sports Nutr Exerc Metab. (2014) 24:360–72. doi: 10.1123/ijsnem.2014-0015 24903758

[12] Alkasasbeh WJ Amawi AT. Impact of eating habits on the psychological state of Jordanian athletes: a descriptive study. Food Sci Technol. (2023) 11:168–81. doi: 10.13189/fst.2023.110305

[13] Bm M Rf O Aj C Ab C Ab C. Summer league college baseball players: do dietary intake and barriers to healthy eating differ between game and non-game days? SMART J. (2007) 3:23–34.

[14] Paschoal VCP Amancio OMS. Nutritional status of Brazilian elite swimmers. Int J Sport Nutr Exerc Metab. (2004) 14:81–94. doi: 10.1123/ijsnem.14.1.81, PMID: 15129932

[15] Evans CEL Christian MS Cleghorn CL Greenwood DC Cade JE. Systematic review and meta-analysis of school-based interventions to improve daily fruit and vegetable intake in children aged 5 to 12 y. Am J Clin Nutr. (2012) 96:889–901. doi: 10.3945/ajcn.111.030270, PMID: 22952187

[16] Reading KJ McCargar LJ Marriage BJ. Adolescent and young adult male hockey players: nutrition knowledge and education. Can J Diet Pract Res. (1999) 60:166–9. PMID: 11551328

[17] Doyle-Lucas AF Davy BM. Development and evaluation of an educational intervention program for pre-professional adolescent ballet dancers: nutrition for optimal performance. J Dance Med Sci. (2011) 15:65–75. doi: 10.1177/1089313X1101500203 21703095

[18] Jagim AR Fields JB Magee M Kerksick C Luedke J Erickson J. The influence of sport nutrition knowledge on body composition and perceptions of dietary requirements in collegiate athletes. Nutrients. (2021) 13:2239. doi: 10.3390/nu13072239, PMID: 34209814 PMC8308384

[19] Posthumus L Fairbairn K Darry K Driller M Winwood P Gill N. Competition nutrition practices of elite male professional rugby union players. Int J Environ Res Public Health. (2021) 18:5398. doi: 10.3390/ijerph18105398, PMID: 34070155 PMC8158491

[20] Hanson J Dole A. Potential nutrition contributions to exercise-associated muscle cramping in four recreational half-marathoners: a case series. Int J Strength Cond. (2021) 1:1–9. doi: 10.47206/ijsc.v1i1.43

[21] Condo D Lohman R Kelly M Carr A. Nutritional intake, sports nutrition knowledge and energy availability in female Australian rules football players. Nutrients. (2019) 11:971. doi: 10.3390/nu11050971, PMID: 31035346 PMC6567108

[22] Ascensión MD. Alimentación y valoración del estado nutricional de los adolescentes españoles (Estudio AVENA). Evaluación de riesgos y propuesta de intervención. I. Descripción metodológica del proyecto* The AVENA group** Conclusión: Este proyecto incluye la actividad coordi-NTS (AVENA Study). Assessment Of Risks And Intervention Proposal. Nutr Hosp. (2003) 1:15–28. 12621808

[23] Cotugna N Vickery CE McBee S. Sports nutrition for young athletes. J Sch Nurs. (2005) 21:323–8. doi: 10.1177/10598405050210060401 16419340

[24] Kunkel ME Bell LB Luccia BH. Peer nutrition education program to improve nutrition knowledge of female collegiate athletes. J Nutr Educ. (2001) 33:114–5. doi: 10.1016/s1499-4046(06)60175-9, PMID: 12031192

[25] Amawi A Alkasasbeh W Jaradat M Almasri A AlObaidi S Abuhammad A. Athletes’ nutritional demands: a narrative review of nutritional requirements. Front Nutr. 10:1331854. doi: 10.3389/fnut.2023.1331854 PMC10848936 38328685

[26] Elgar FJ Roberts C Tudor-Smith C Moore L. Validity of self-reported height and weight and predictors of bias in adolescents. J Adolesc Health. (2005) 37:371–5. doi: 10.1016/j.jadohealth.2004.07.014, PMID: 16227121

[27] Kuczmarski RJ. 2000 CDC growth charts for the United States: methods and development. Vital Health Stat. (2002) 246:1–190. 12043359

[28] Andajani-Sutjahjo S Ball K Warren N Inglis V Crawford D. Perceived personal, social and environmental barriers to weight maintenance among young women: a community survey. Int J Behav Nutr Phys Act. (2004) 1:15. doi: 10.1186/1479-5868-1-15, PMID: 15462679 PMC524367

[29] Vázquez-Espino K Fernández-Tena C Lizarraga-Dallo MA Farran-Codina A. Development and validation of a short sport nutrition knowledge questionnaire for athletes. Nutrients. (2020) 12:1–14. doi: 10.3390/nu12113561 PMC7699674 33233681

[30] Brauman K Achen R Barnes JL. The five most significant barriers to healthy eating in collegiate student-athletes. J Am Coll Health. (2023) 71:578–83. doi: 10.1080/07448481.2021.1899186 33830870

[31] Spronk I Kullen C Burdon C O’Connor H. Relationship between nutrition knowledge and dietary intake. Br J Nutr. (2014) 111:1713–26. doi: 10.1017/S0007114514000087 24621991

[32] Debnath M Chatterjee S Bandyopadhyay A Datta G Dey SK. Prediction of athletic performance through nutrition knowledge and practice: a cross-sectional study among young team athletes. Sport Mont. (2019) 17:13–20. doi: 10.26773/smj.191012

[33] Noronha DC Santos MIAF Santos AA Corrente LGA Fernandes RKN Barreto ACA. Nutrition knowledge is correlated with a better dietary intake in adolescent soccer players: a cross-sectional study. J Nutr Metab. (2020) 2020:1–7. doi: 10.1155/2020/3519781, PMID: 31998535 PMC6964714

[34] Jusoh N Low Fook Lee J Tengah RY Azmi SH Suherman A. Association between nutrition knowledge and nutrition practice among Malaysian adolescent handball athletes. Malays. J Nutr. (2021) 27:27. doi: 10.31246/mjn-2020-0113

[35] Kosendiak A Król M Ligocka M Kepinska M. Eating habits and nutritional knowledge among amateur ultrarunners. Front Nutr. (2023) 10:10. doi: 10.3389/fnut.2023.1137412 PMC10365969 37497055

[36] Tam R Beck KL Manore MM Gifford J Flood VM O’Connor H. Effectiveness of education interventions designed to improve nutrition knowledge in athletes: a systematic review. Sports Med. (2019) 49:1769–86. doi: 10.1007/s40279-019-01157-y, PMID: 31372860

[37] Foo WL Faghy MA Sparks A Newbury JW Gough LA. The effects of a nutrition education intervention on sports nutrition knowledge during a competitive season in highly trained adolescent swimmers. Nutrients. (2021) 13:2713. doi: 10.3390/nu13082713, PMID: 34444873 PMC8400374

[38] Crites SL Aikman SN. Impact of nutrition knowledge on food evaluations. Eur J Clin Nutr. (2005) 59:1191–200. doi: 10.1038/sj.ejcn.1602231 16015254

[39] Krešić G Kenđel Jovanović G Pavičić Žeželj S Cvijanović O Ivezić G. The effect of nutrition knowledge on dietary intake among Croatian university students. Coll Antropol. (2009) 33:1047–56. PMID: 20102047

[40] Judge LW Kumley RF Bellar DM Pike KL Pierson EE Weidner T. Hydration and fluid replacement knowledge, attitudes, barriers, and behaviors of NCAA division 1 American football players. J Strength Cond Res. (2016) 30:2972–8. doi: 10.1519/JSC.0000000000001397, PMID: 26950346

[41] Alawamleh T AlKasasbeh W. Exploring the landscape of eHealth in promoting physical activity and healthy dietary intake. Univers J Public Health. (2024) 12:120–7. doi: 10.13189/ujph.2024.120113

[42] Andrews A Wojcik JR Boyd JM Bowers CJ. Sports nutrition knowledge among mid-major division I university student-athletes. J Nutr Metab. (2016) 2016:1–5. doi: 10.1155/2016/3172460, PMID: 27872757 PMC5107837

[43] Ahmadi F Ebrahimi M Kashani V. Sports nutritional knowledge, attitude, and practice of adolescent athletes in Tehran, Iran. Asian. J Sports Med. (2022) 13:e131584. doi: 10.5812/asjsm-131584

[44] Bakhtiar M Masud-ur-Rahman M Kamruzzaman M Sultana N Rahman SS. Determinants of nutrition knowledge, attitude and practices of adolescent sports trainee: a cross-sectional study in Bangladesh. Heliyon. (2021) 7:e06637. doi: 10.1016/j.heliyon.2021.e06637, PMID: 33898807 PMC8056407

[45] Philippou E Middleton N Pistos C Andreou E Petrou M. The impact of nutrition education on nutrition knowledge and adherence to the Mediterranean diet in adolescent competitive swimmers. J Sci Med Sport. (2017) 20:328–32. doi: 10.1016/j.jsams.2016.08.023, PMID: 27692575

[46] White HJ Harwood CG Wiltshire G Plateau CR. Parents’ experiences of family food routines in adolescent elite-level swimming. Psychol Sport Exerc. (2022) 62:102237. doi: 10.1016/j.psychsport.2022.102237

[47] O’brien L Collins K Amirabdollhian F. Exploring sports nutrition knowledge in elite gaelic footballers. Nutrients. (2021) 13:1081. doi: 10.3390/nu13041081 33810237 PMC8066959

[48] AlKasasbeh WJ Tawfiq AA. The effectiveness of using Mobile learning application on undergraduates’ intrinsic motivation and their general nutrition knowledge. Int J Interact Mob Technol. (2023) 17:19–37. doi: 10.3991/ijim.v17i17.40959

[49] Scarry A Rice J O’connor EM Tierney AC. Usage of Mobile applications or Mobile health technology to improve diet quality in adults. Nutrients. (2022) 14:2437. doi: 10.3390/nu14122437 35745167 PMC9230785

[50] Vasiloglou MF Christodoulidis S Reber E Stathopoulou T Lu Y Stanga Z. What healthcare professionals think of ″nutrition & diet″ apps: an international survey. Nutrients. (2020) 12:1–17. doi: 10.3390/nu12082214 PMC7468977 32722339

